# Distinct Superficial and Deep Laminar Domains of Activity in the Visual Cortex during Rest and Stimulation

**DOI:** 10.3389/fnsys.2010.00031

**Published:** 2010-08-10

**Authors:** Alexander Maier, Geoffrey K. Adams, Christopher Aura, David A. Leopold

**Affiliations:** ^1^Unit on Cognitive Neurophysiology and Imaging, Laboratory of Neuropsychology, National Institute of Mental Health, National Institutes of HealthBethesda, MD, USA; ^2^Neurophysiology Imaging Facility, National Institute of Mental Health, National Institute of Neurological Disorder and Stroke, National Eye Institute, National Institutes of HealthBethesda, MD, USA

**Keywords:** resting state, V1, laminae, gamma, LFP, coherence, layers, visual perception

## Abstract

Spatial patterns of spontaneous neural activity at rest have previously been associated with specific networks in the brain, including those pertaining to the functional architecture of the primary visual cortex (V1). However, despite the prominent anatomical differences between cortical layers, little is known about the laminar pattern of spontaneous activity in V1. We address this topic by investigating the amplitude and coherence of ongoing local field potential (LFP) signals measured from different layers in V1 of macaque monkeys during rest and upon presentation of a visual stimulus. We used a linear microelectrode array to measure LFP signals at multiple, evenly spaced positions throughout the cortical thickness. Analyzing both the mean LFP amplitudes and between-contact LFP coherences, we identified two distinct zones of activity, roughly corresponding to superficial and deep layers, divided by a sharp transition near the bottom of layer 4. The LFP signals within each laminar zone were highly coherent, whereas those between zones were not. This functional compartmentalization was found not only during rest, but also when the receptive field was stimulated during a visual task. These results demonstrate the existence of distinct superficial and deep functional domains of coherent LFP activity in V1 that may reflect the intrinsic interplay of V1 microcircuitry with cortical and subcortical targets, respectively.

## Introduction

There is abundant activity in the brain in the absence of explicit sensory input or behavioral output, a phenomenon that is commonly observed in both electrophysiological and brain imaging experiments. In fact, most of the brain’s enormous energy expenditure appears to arise from spontaneously driven, intrinsic processes, rather than from the interaction with the sensory environment. Sensory stimulation increases local energy consumption above this background of high metabolic activity by only a few percent (Clarke and Sokoloff, 1999; Shulman et al., 2004; Raichle and Mintun, 2006). Yet despite its prominence, the neural processes associated with this spontaneous ongoing activity (SOA) have not been examined in detail until recently, and their significance for normal brain function is poorly understood. Moment-by-moment fluctuations in neural activity that cannot be ascribed to a stimulus or task event are typically treated as random “noise,” and are thus averaged out over multiple experimental trials.

Analyzing spontaneous neural activity poses certain experimental challenges, as there are no clearly defined task events to serve as points about which to average. A common approach has therefore been to investigate the temporal covariation between pairs of signals simultaneously measured at different positions in the brain. Approximately 15 years ago, this approach was applied in two different branches of experimental neuroscience – functional magnetic resonance imaging (fMRI) in humans, and electrophysiology in anesthetized cats – with results in each case demonstrating that SOA exhibits reproducible spatiotemporal patterns that can be linked to underlying neural circuits. Using fMRI, Biswal et al. (1995) demonstrated that spontaneous activity within a functional sensorimotor network showed strong covariation even when that network was completely at rest, a phenomenon they dubbed “functional connectivity” based largely on previous electrophysiological work (Gerstein and Perkel, 1969; Gochin et al., 1991; Friston, 1994). In the same year, Arieli et al. (1995) used electrophysiological and optical techniques to show that patterns of intrinsic electrical activity in the visual cortex of anesthetized cats is coordinated at spatial scales up to several millimeters.

Over the next decade, fMRI studies in humans and electrophysiological studies in animals elaborated the degree of organization of SOA signals. Functional connectivity computed from fMRI collected during rest revealed multiple distinct “networks” of covarying (i.e., functionally connected) areas (for a review, see Fox and Raichle, 2007). Early studies focused on cortical networks (Lowe et al., 1998; Greicius et al., 2003; Fox et al., 2005), with more recent ones also demonstrating subcortical networks (Di Martino et al., 2008; Zhang et al., 2008; O’Reilly et al., 2010). Relatively few imaging studies have been conducted in animal models to date, but the basic pattern of resting state networks appears similar in monkeys and humans (Vincent et al., 2007; Moeller et al., 2009). While there remain questions as to the neural basis and functional significance of these covarying networks, they have been consistently observed under a variety of conditions, drawing considerable attention to the neural underpinnings of SOA (He et al., 2008; Nir et al., 2008; Shmuel and Leopold, 2008; Hayden et al., 2009; Schölvinck et al., 2010). In parallel with fMRI studies, some electrophysiological studies applied the covariation approach to characterize spontaneous signals in animals. For example, Arieli and colleagues extended their original work to show that the spatial map of covariation between the firing rate of neurons and the membrane potentials measured optically over several millimeters closely resembled the functional architecture of orientation columns measured in separate experiments from the same patch of cortex (Tsodyks et al., 1999; Kenet et al., 2003). These findings demonstrated that SOA in V1 is functionally organized across the cortical surface, possibly due to the pattern of horizontal connections known to exist between orientation columns.

One of the most prominent anatomical features of the cerebral cortex is its laminar organization, with individual cortical layers differing substantially in cell types and projection patterns. This dimension is perpendicular to the cortical surface, and therefore currently inaccessible with optical imaging techniques. As a consequence, spatial patterns of SOA across layers are still largely unexplored. One early study, coincidentally published in the same year as the seminal fMRI and optical imaging studies mentioned above, examined differences in spontaneous neural firing rate in area V1 of the awake monkey as a function of layer (Snodderly and Gur, 1995). Mean firing rates differed substantially across the cortical thickness, with cells in layers that receive thalamic input having higher intrinsic firing rates compared to those in other layers. In that study, single electrodes were used to measure activity from one position at a time, so the temporal covariation between layers could not be evaluated.

Using linear multicontact electrode arrays, it has become possible to simultaneously record neural activity at equally spaced intervals across all cortical layers, from the *pia mater* to the white matter. This approach is often used to study the laminar profile of local field potential (LFP) responses in V1 to visual stimulation (Mitzdorf, 1985; Schroeder et al., 1991), whose depth and timing can then be related to the underlying anatomy. However, to date few if any studies have examined how the SOA of field potentials covaries between different layers. Here we investigate the laminar structure of spontaneous neural signals in the primary visual cortex of macaque monkeys by evaluating their temporal correlation as a function of laminar position. Specifically, we ask three fundamental questions. First, how does the amplitude of the LFP signal vary spatially as a function of cortical depth? Second, between which layers do LFP signals display high degrees of temporal coherence? And third, to what extent are the specific patterns of SOA influenced by the presentation of a visual stimulus to a neuron’s receptive field?

We report that spontaneous LFP activity in V1 varies significantly as a function of cortical layer, with prominent differences between a superficial compartment (layers 1–4) and a deep compartment (layers 5 and 6). The magnitude of SOA fluctuations in the gamma-range (30–100 Hz) was roughly twice as large in the superficial compartment compared to a deep one. Moreover, the temporal coherence of signals within each zone was substantially stronger than that between zones, with an abrupt discontinuity near the bottom of layer 4. Finally, these laminar patterns were observed during both quiet rest and active stimulation during a visual task. We speculate that this functional compartmentalization of SOA into superficial and deep laminar zones reflects the interplay of V1 with cortical and subcortical targets, respectively.

## Materials and Methods

### Subjects

Two healthy adult male monkeys (*Macaca mulatta*), 98X009 and CB35, were used in the study. All procedures followed US National Institutes of Health guidelines, were approved by the Animal Care and Use Committee of the US National Institute of Mental Health and were conducted with great care for the comfort and well being of the animals.

### Surgery

Monkeys were implanted under sterile conditions and isoflurane anesthesia (1.5–2%) with custom-designed and fabricated fiberglass headposts, fixed to the skull using transcranial ceramic screws (Thomas Recording, Giessen, Germany), Copalite Varnish (Colley and Colley, Ltd., Houston, TX, USA), and self-curing denture acrylic (Lang Inc., Wheeling, IL, USA). In a subsequent surgery, a recording chamber was implanted over V1 using frameless stereotaxy guided by high-resolution anatomical magnetic resonance images (Brainsight, Rogue Research), and a craniotomy was performed inside the chamber. Animals received antibiotics and analgesics post-operatively.

### Experimental Paradigm

There were two conditions evaluated in the main portion of the study: rest and visual stimulation, as well as a third condition (flashing screen) used to generate the current source density (CSD) profile of each session (Figure S1 in Supplementary Material). Ambient light in the testing room was minimized, though not completely absent. In all cases, the three conditions were collected during the same session, with the electrode in the same place. During the *rest* condition, activity was recorded over a 20-min period during which animals sat alone in a darkened quiet room, with the monitors turned off. The animals were free to move their eyes about, and frequently closed their eyes for brief periods. The *visual stimulation* condition was intended mainly as a behavioral contrast to the resting condition. The monkey was required to maintain its gaze upon a very small dot (0.1 dva) appearing in the middle of a dark screen for periods lasting 5.3 s, during which time visual stimuli were presented away from the fixation spot. The stimuli consisted of a static disk in the receptive field region followed by a surrounding field of moving random dots, with the precise stimulus sequence described elsewhere (Maier et al., 2008). Note that during visual stimulation the monkeys were required to fixate within a window of up to 2 dva radius and receiving a juice reward upon completion of each trial. If a monkey broke fixation, the trial was aborted and re-initiated after a short delay of 100–800 ms. The animal’s eye movements were monitored and recorded using an infrared light sensitive camera and commercially available eye tracking software (Eye Link II, RS Research, Osgoode, Canada). Finally, each session contained a 5–10 min testing period in which the monkey was repeatedly stimulated with a full screen, flashing stimulus. This stimulation was used *post hoc* to compute a conventional pattern of CSD responses to visual stimulation (see below). Once each second the screen was turned from black (∼0.2 cd/m^2^) to white (∼130 cd/m^2^) for 100 ms as the monkey fixated near its center.

All visual stimuli were generated using OpenGL-based custom written software (ESS/STIM, courtesy Dr. D. Sheinberg) running on industrial PCs (Kontron, Poway, CA, USA) with NVIDIA Quadro FX 3000 graphics boards. Stimuli were presented on either a single 18′ TFT monitor placed in front of the animals (NEC MultiSync LCD 1860NX with a 1024 × 768 resolution) or two 27′ TFT monitors (X2Gen MV2701, 1024 × 768 resolution) with a diagonal of 32′ (X2Gen MV2701, 1024 × 768 resolution) mounted on opposite walls of the test box at a viewing distance of 80 cm and a custom made mirror stereoscope mounted in front of the head restrained animal.

### Neurophysiological Recordings

Laminar LFP was collected during 13 recording sessions (6 in monkey 98X009). During each session, data were recorded under three different conditions (1) viewing a flashing visual screen, used to compute the CSD used to identify layer 4, (2) executing a simple fixation task while being presented visual stimuli, and (3) sitting quietly in a dimly lit room with no explicit task or stimulus (see below). Recordings were performed inside an RF-shielded booth. LFP (defined as extracellular voltage fluctuations in the frequency range between 1 and 100 Hz) were recorded from primary visual cortex in all animals. All recording sites were from dorsal V1, several millimeters posterior to the lunate sulcus, in the parafoveal region of the visual field (see Figure S2A in Supplementary Material).

Recordings were performed using a 24-contact microelectrode with an inter-contact spacing of 100 μm (Neurotrack Ltd, Békéscsaba, Hungary), with contact impedances varying between 0.3 and 0.5 MΩ. The multicontact electrode was manually lowered into cortex using a custom designed microdrive and signals were amplified and recorded using the Plexon MAP system (Plexon Inc., Dallas, TX, USA), with the shank of the electrode serving as both the grounding point and the electrical reference. Coarse positioning of the electrode was achieved by monitoring the visually evoked potential during the flashing screen paradigm. Specifically, the electrode position was adjusted according to the position of the polarity reversal of response to the flash (see Figure S3 in Supplementary Material for intersession accuracy of the electrode placement). Additional alignment, based on the CSD computed offline, was done prior to averaging across sessions (see below).

### Data Analysis

All neurophysiological data were processed and analyzed using custom written code in MATLAB. The LFP was filtered between 1 and 200 Hz, amplified by a factor of 1000 and digitized at 1 kHz for data collection, and subsequently down-sampled to 250 Hz after low-pass filtering with an eighth-order, bi-directional, zero-phase Chebyshev type-1 filter with a cutoff frequency of 100 Hz. This provided a time-varying voltage signal for each channel that served as the basis of further analysis. Frequency analysis was performed using a fast Fourier transform algorithm. Magnitude spectra were computed using a modified Welch’s method, with multitaper analysis revealing similar results. The signal was split into Hamming windows of 512 ms length (and 50% overlap). The magnitude of each windowed segment (doubled in signal content to account for negative frequencies as well as normalized using a window-dependent scale factor) was computed, and the results were time-averaged. Power spectral density (PSD) was computed in a similar manner using 256 ms windows, with an additional step of squaring the signal magnitude to obtain the power spectrum.

Coherence estimates were computed as magnitude-squared coherence *C_xy_*(*f*) using Welch’s averaged, modified periodogram method and the following formula:

$$C_{xy}\left(f\right)=\frac{{\left|P_{xy}\left(f\right)\right|}^{2}}{P_{xx}\left(f\right)P_{yy}\left(f\right)}$$

where *P_xx_*(*f*) and *P_yy_*(*f*) are the power spectral densities of each individual signal *x*(*t*) and *y*(*t*), and *P_xy_*(*f*) is their cross PSD. The resulting functions denote the degree of signal correspondence, or coupling, as a function of frequency, with 1 indicating perfect correspondence. All coherence measures were performed by averaging the results computed for overlapping 256 ms epochs and averaged consecutively (Welch’s method).

Band-limited power (BLP) was computed by band-pass filtering the signal using a second-order, bi-directional, zero-phase Chebyshev type-1 band-pass filter (frequency ranges are indicated in the text). Power was computed by full-wave rectifying the band-limited signals. This results in a measure of time-varying amplitude, or signal power (in actuality, the square root of the power), in each frequency band and is roughly equivalent to averaging several adjacent rows of a spectrogram (Leopold et al., 2003).

For approximating the layers corresponding to each session prior to alignment and averaging, we relied on data from the flashing screen condition. For each session, data from at least 100 stimulus presentations was averaged for each electrode contact. We applied CSD analysis to this data *post hoc* using a standard algorithm (based on the second spatial derivative estimate of the laminar LFP time series), as well as the spline-based algorithms of the iCSD toolbox for MATLAB (Pettersen et al., 2006). This analysis revealed a robust short-latency current sink in the middle layers for each session (Figure 3A). Previous studies have shown that this sink in V1 corresponds most closely to layer 4Cα (Mitzdorf and Singer, 1979; Schroeder et al., 1991). We treated the center of this sink as a point of alignment (the “zero point”) for each session, and considered the zone ±200 μm superficial and deep to this reference to be the approximate extent of layer 4, though the results do not critically depend on this approximation. Note that due to this procedure fewer sessions contribute to the shallowest and deepest “adjusted relative depths,” although we limited the overall extent of our analysis to ±1000 μm from zero, thus restricting the analysis to depths where the majority of sessions contributed.

## Results

**Figure 1:**
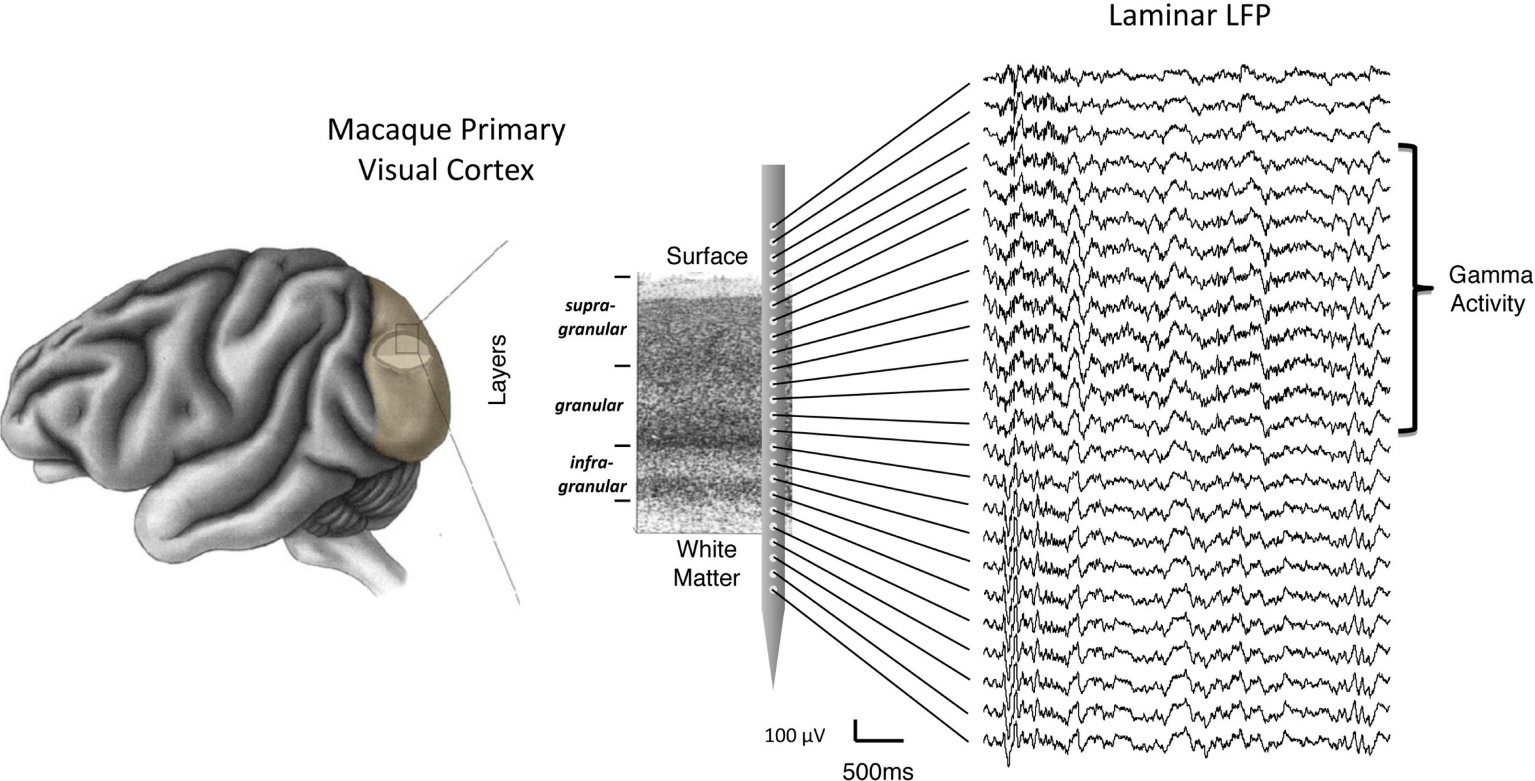
**Schematic representation of laminar LFP recordings from a linear multicontact electrode array in the primary visual cortex**. For illustrative purposes, the array is depicted overlying a Nissl-stained histological slice, with labels showing individual layers and the corresponding compartments (i.e., supragranular, granular, and infragranular) used in the study. On the right is a sample of extracellular LFP data collected in one experiment. Each trace corresponds to the voltage measured simultaneously as a function of time (see scale bar). In this example, the top contacts span an area reaching from outside the brain (top) to the white matter (bottom). Note the spatial non-uniformity of gamma frequency signals, superimposed on the signals, and restricted to the upper cortical layers.

**Figure 2:**
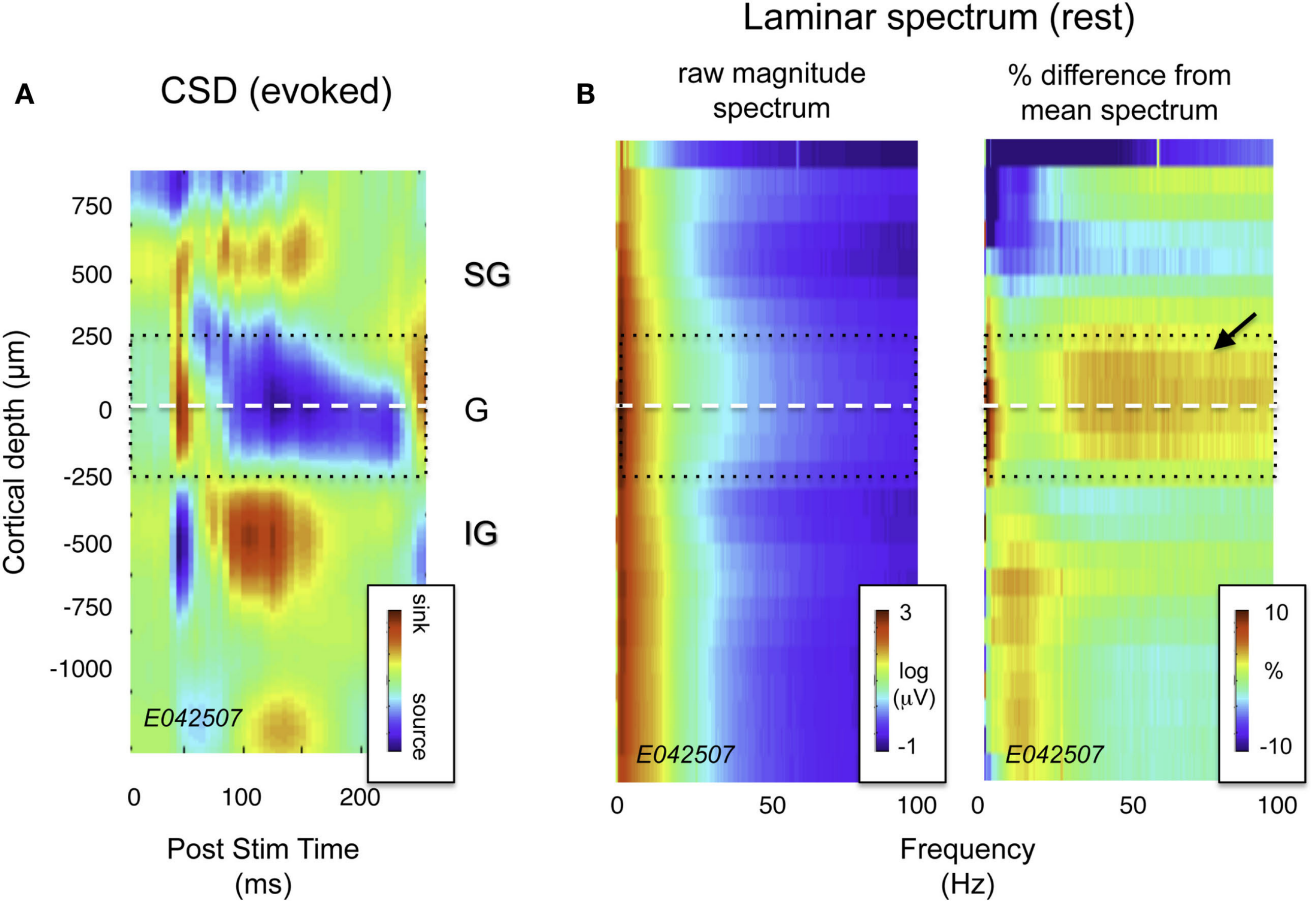
**Current source density (CSD) and spectral profile of LFP magnitude as a function of laminar position for one session**. **(A)** CSD profile in response to a flickering screen collected during an example session (monkey CB35). The center of the initial current sink, thought to correspond primarily to thalamic input in layer 4Cα, was used as a point of alignment throughout the study. Dotted lines ±250 μm correspond roughly to the extent of layer 4. **(B)** Spectral laminar profile during rest. On the left is a plot depicting mean LFP magnitude for a representative 20-min period following the CSD experiment with the monkey at rest. Signal magnitude is color-coded on a log scale, and plotted as a function of frequency and cortical depth. On the right is the same data, normalized by the mean spectrum across all layers, and expressed as percent deviation from this mean. Note the elevated LFP magnitude in the granular and deep supragranular layers (arrow). This feature proved highly consistent across sessions (see Figure 3A).

The laminar properties of the LFP were evaluated during 13 sessions in two monkeys while they were either at rest in a dark room or while they were actively performing a visual task (see Materials and Methods). At the beginning of each session a linear multicontact electrode array (Figure 1) was inserted perpendicular to the cortical surface of V1 and advanced 2 mm with the monkey at rest. The LFP signal was recorded in parallel from 24 electrode contacts at equally spaced intervals (100 μm) spanning from the *pia mater* to the white matter. The pattern of CSD responses to a flashing stimulus (see Materials and Methods) collected at the beginning of each session was used *post hoc* to establish the spatial positions of individual electrode contacts relative to specific cortical laminae (see Figures S1A and S2 in Supplementary Material). To verify the stability of the electrode positioning, we also sometimes collected the CSD profile a second time, at the end of the session (see Figure S5C in Supplementary Material). This method of anatomical registration is based on previous work in the primary visual cortex of monkeys employing a combination of CSD analysis, microlesions, and *post mortem* histology, which demonstrated that the initial current sink originates in layer 4C, possibly with its peak in layer 4Cα (Mitzdorf and Singer, 1979; Schroeder et al., 1991). We thus took the initial sink as the primary point of alignment, and used this alignment as the basis for averaging data over many sessions. Specifically, we aligned each day’s data by centering the LFP traces of the 24 electrode contacts around the initial current sink (see Figure S2 in Supplementary Material). This created a new reference frame with its zero point located in the middle of layer 4. Then starting from the zero point we coarsely divided the cortex into supragranular (SG, layers 1–3), granular (G, layer 4), and infragranular (IG, layers 5 and 6) zones. The boundaries of these zones, defined as ±250 μm (corresponding to an inclusion criterion of two channels above and below the one upon which we centered the data) are intended only as an approximate reference for the upper and lower bounds of layer 4 (although it did match the extent of the initial sink notably well; see Figure 2A).

## Predominance of Gamma Activity in Upper Layers

Visual inspection of the raw LFP traces during each session (e.g., Figure 1) revealed that certain temporal features in the SOA were shared by only a subset of channels. Notably, there was a stripe of low amplitude, high-frequency activity superimposed on the signals in the more superficial channels. This can be seen clearly in Figure 2B, which shows the spectral analysis of a single session as a function of cortical depth. For reference, Figure 2A shows the CSD profile obtained from the flashing screen condition used for laminar alignment during the same session. In this example, there is an elevation of high-frequency LFP activity (roughly 30–100 Hz) in the G and deep SG layers, as established from the CSD profile.

**Figure 3:**
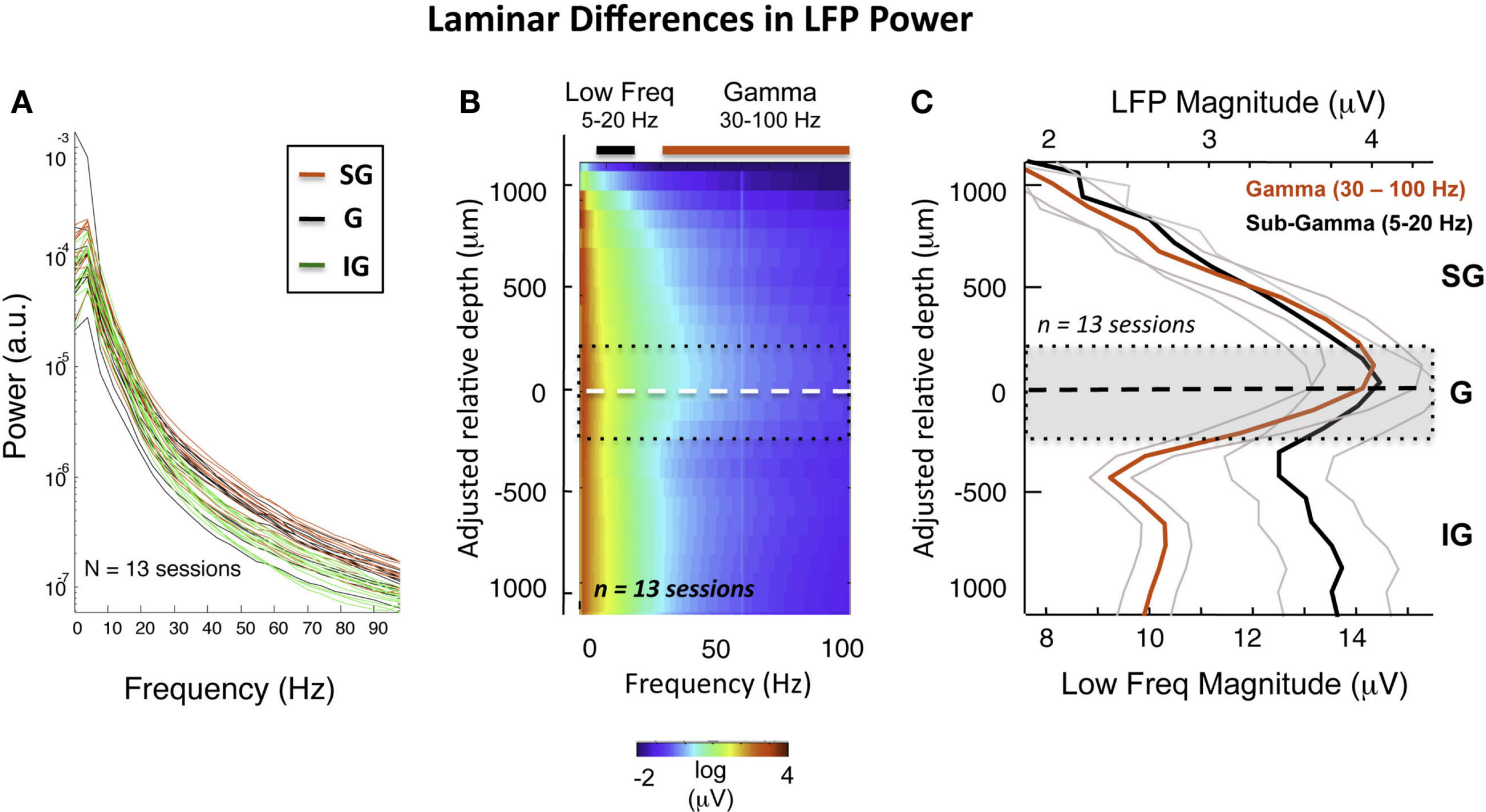
**Intersession average following CSD-based realignment**. **(A)** Laminar differences as a function of frequency over all sessions. **(B)** The mean LFP magnitude during resting state over all sessions following alignment, expressed as a function of frequency. The dashed white line represents the “zero point,” with the dotted black lines showing rough boundaries of layer 4. The elevated high-frequency activity in the middle and upper layers is clearly visible. The colored bars indicate the frequency range used to compute mean LFP magnitude in **(C)**. **(C)** Laminar distribution of LFP amplitude in gamma and sub-gamma-ranges during rest. Note that the mean gamma-range amplitude is highest in the middle and upper layers.

This general pattern was observed across all sessions and V1 sites in two monkeys (see Figure S3 in Supplementary Material for individual sessions). We quantified these spectral differences by calculating the PSD of the LFP for each of the three main laminar compartments. Figure 3A plots the PSD averaged across 20 min with the monkeys at rest, on a session-by-session basis (see Materials and Methods). Each line represents the power spectrum of one session, color-coded for signal origin (red = SG; black = G; green = IG). For frequencies above ∼30 Hz, the infragranular LFP showed considerably lower power than the supragranular LFP (note the log scale). This pattern proved highly consistent across recording sessions in both animals.

Based on the alignment of electrode contacts described above (see Figure S4 in Supplementary Material), data from individual sessions could thus be brought into correspondence, estimated to be within 200 μm (see Figure S5 in Supplementary Material). This allowed us to compute the averaged laminar magnitude spectrum (Figure 3B) over all sessions. Figure 3C shows the resulting laminar profile of ongoing LFP power in the gamma (30–100 Hz) and sub-gamma (5–20 Hz) frequency ranges. In line with the pattern revealed by the session-by-session comparison discussed above, we found that, on average, gamma power was roughly 50% higher in the G and SG zone than in the IG zone across the population (red curve). This difference was not present in the sub-gamma-range (black curve).

What might be the basis of the laminar differences in gamma-range power? One possibility is that superficial and deep layers participate in different aspects of the brain’s intrinsic activity during the resting state. This conjecture is consistent with the known anatomical segregation of neural afferents, differences in cell types, projection targets, and other aspects of the laminar anatomy (see Discussion). To address whether superficial and deep layers differ in their pattern of functional interactions, we next investigated the laminar covariation of SOA by computing the temporal coherence between all pairs of electrodes.

## Superficial and Deep Zones of LFP Coherence

**Figure 4:**
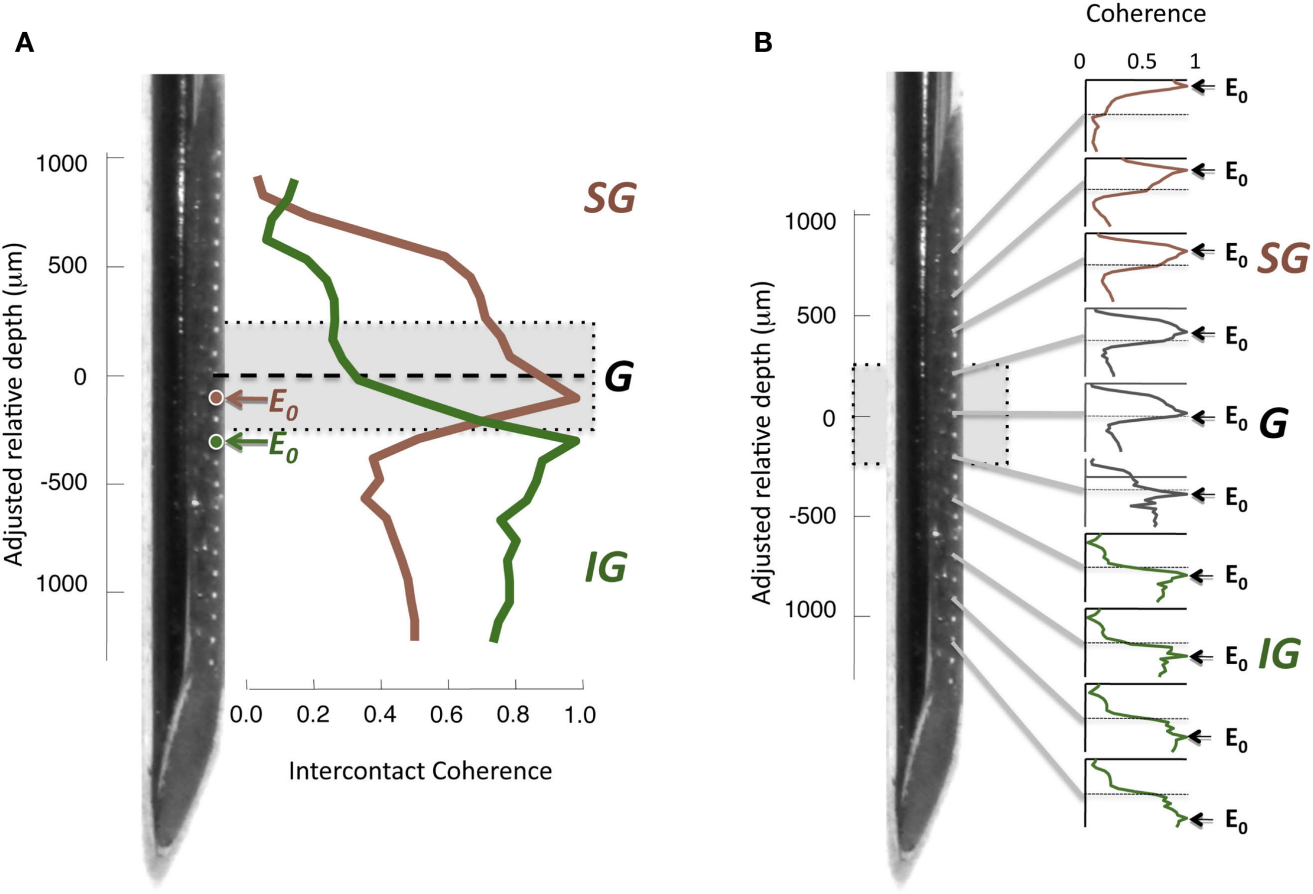
**(A) Inter-contact coherence for reference contacts taken from two different laminar compartments**. Magnitude-squared coherence in the gamma-range (30–100 Hz) is plotted between each reference electrode and all other electrodes in the array (including contacts located in the white matter at the bottom of the array and outside the brain at the top, respectively). The electrode from the granular zone (red) showed strong correlation with granular and supragranular sites, but weaker coherence with infragranular sites. In contrast, the infragranular contact (green) showed strong coherence with the infragranular contacts, but very low coherence with other sites. Note that the two electrodes chosen for this example (E_0_, red and green) are separated by only 200 μm. **(B)** Laminar pattern of inter-contact coherence for 10 different E_0_ contact positions, shown in the same format as in **(A)**. The E_0_ positions in the infragranular layers elicit a pattern of high coherence only in those layers, suggesting that the gamma-range activity in those layers is highly synchronous, but asynchronous to that in other layers. Conversely, E_0_ positions in the supragranular layers are coherent only with signals measured in supragranular contacts. A single contact lying just below the zero point appears to be a transition between supra- and infragranular coherence.

To assess the interdependence of the LFP time course in different cortical layers, we computed the magnitude-squared coherence between signals measured from different electrode contacts. Coherence is a measure of similarity in the temporal structure of two signals that quantifies the extent to which two signals are linearly related. A coherence value equal to zero indicates that there is no consistent relationship between the two signals, whereas a value of 1 indicates there is a perfectly linear relationship. For each electrode contact, a spatial profile of coherence can be determined by computing the magnitude-squared coherence between its time course and the time course of the contacts at the other spatial positions. An example of this approach from one session in the resting condition is shown in Figure 4A, with coherence pertaining to LFP frequencies in the gamma-range (30–100 Hz). In this figure, two spatial profiles were computed, one for a contact in the G layer, and one for a contact in the IG layers. Note that the contact in the G layer (E_0_, red) showed strong coherence with the other G positions and most of the SG positions, but the coherence level fell abruptly in the IG layers. In contrast, the IG contact (E_0_, green), situated a mere 200 μm deeper, showed the opposite pattern. Its highest coherence was with the deeper electrodes, whereas it showed minimal coherence with the superficial electrodes. This analysis is expanded in the same session in Figure 4B, which shows the laminar coherence profile for 10 different contacts. Pairs of electrodes in the G and SG layers show high coherence in the gamma-range, as do pairs of IG electrodes. However coherence between compartments is much lower, suggesting distinct processes in the upper and lower layers in the gamma-range.

**Figure 5:**
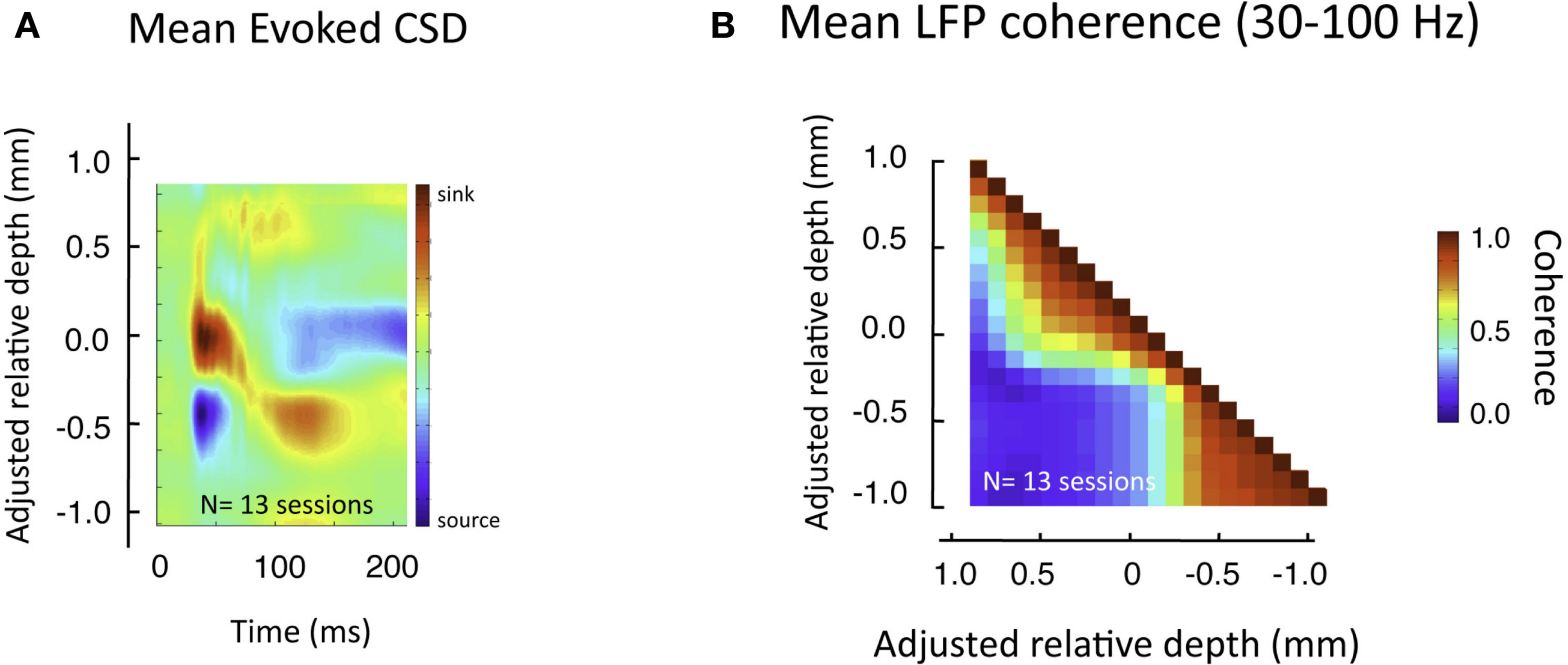
**Pair-wise coherence of all sessions in the gamma-range**. **(A)** Average CSD to flashing screen following alignment to initial sink. **(B)** Mean gamma-range coherence, computed between all pairs of laminar positions over all sessions, during rest (see Figure 7 for effects of visual stimulation). The red regions reveal the high inter-compartmental coherence, with the adjacent blue regions revealing the lack of coherence between compartments.

**Figure 6:**
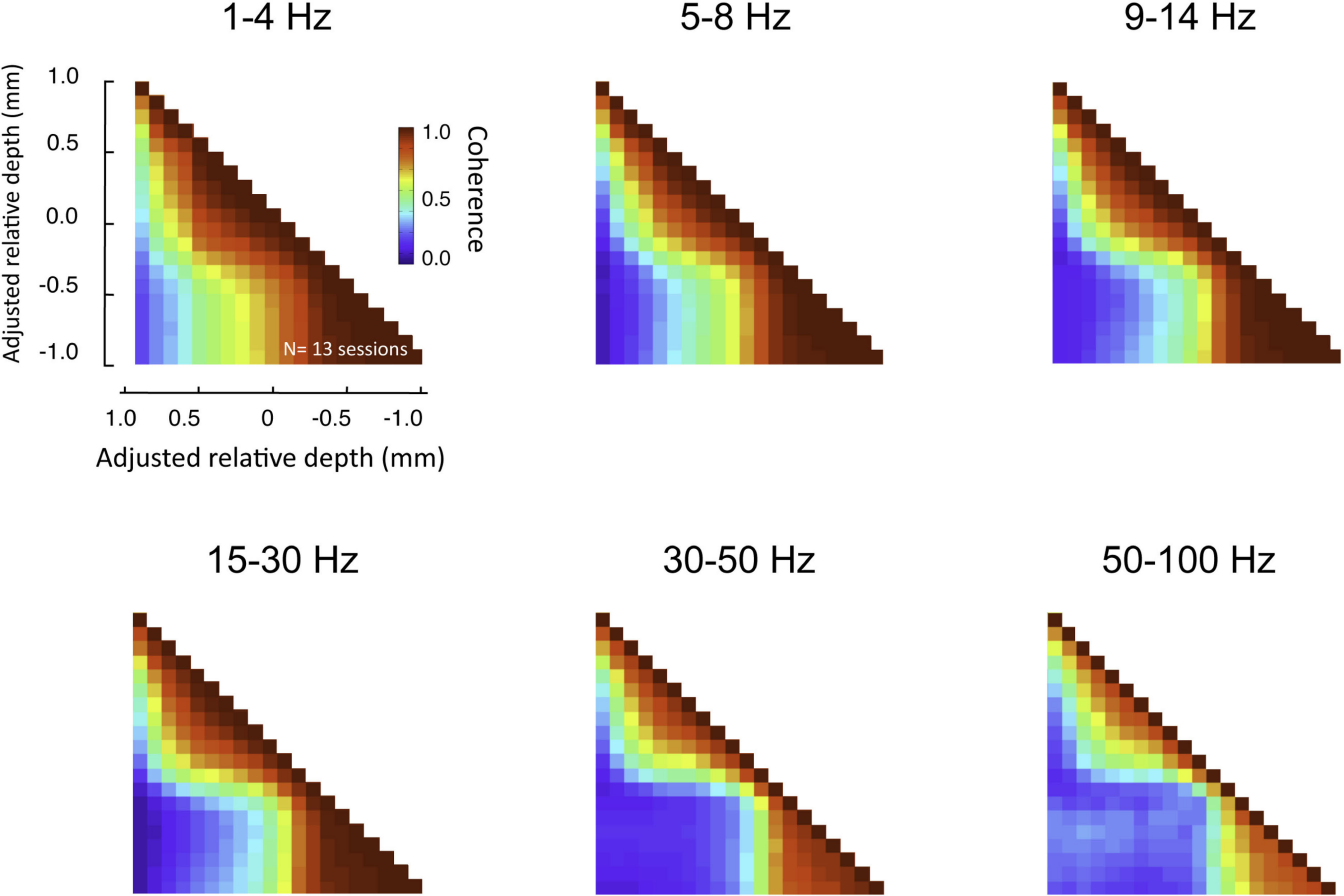
**Laminar coherence as a function of frequency (n = 13, session; both monkeys)**. Inter-compartmental coherence for the classic EEG frequency bands (delta = 1–4 Hz, theta = 5–8 Hz, alpha = 9–14 Hz, beta = 15–30 Hz, low gamma = 30–50 HZ, high gamma = 50–100 Hz) is plotted individually using the same format as Figure 7. Note that despite differences in the overall coherence, the basic pattern between upper and lower layers remained.

This pattern of laminar coherence was consistent across recording sessions and animals. The population pair-wise coherence in the (30–100 Hz) gamma-range is depicted in Figure 5B, adjacent to the mean aligned CSD response for all sessions (Figure 5A). Each square in the matrix corresponds to the mean gamma-range coherence value over all sessions, relative to the zero-point alignment contact. The large, red regions reflect the strong similarity of signals measured within the same laminar compartment, whereas the blue background reflects the fact that the between-compartment coherence is weak. Note that due to the alignment, the transition between the two compartments is abrupt, even when averaged over all sessions, indicating a sharp discontinuity between zones of coherent activity. The data from each session, shown separately in Figure S3 in Supplementary Material, demonstrates the day-to-day consistency of these main findings. Figure 6 shows the same analysis for other frequency ranges. In contrast to LFP amplitude, which showed significant laminar differences in the gamma-range only, LFP coherence of almost all frequencies segregated significantly between laminar compartments.

## Effects of Visual Stimulation

**Figure 7:**
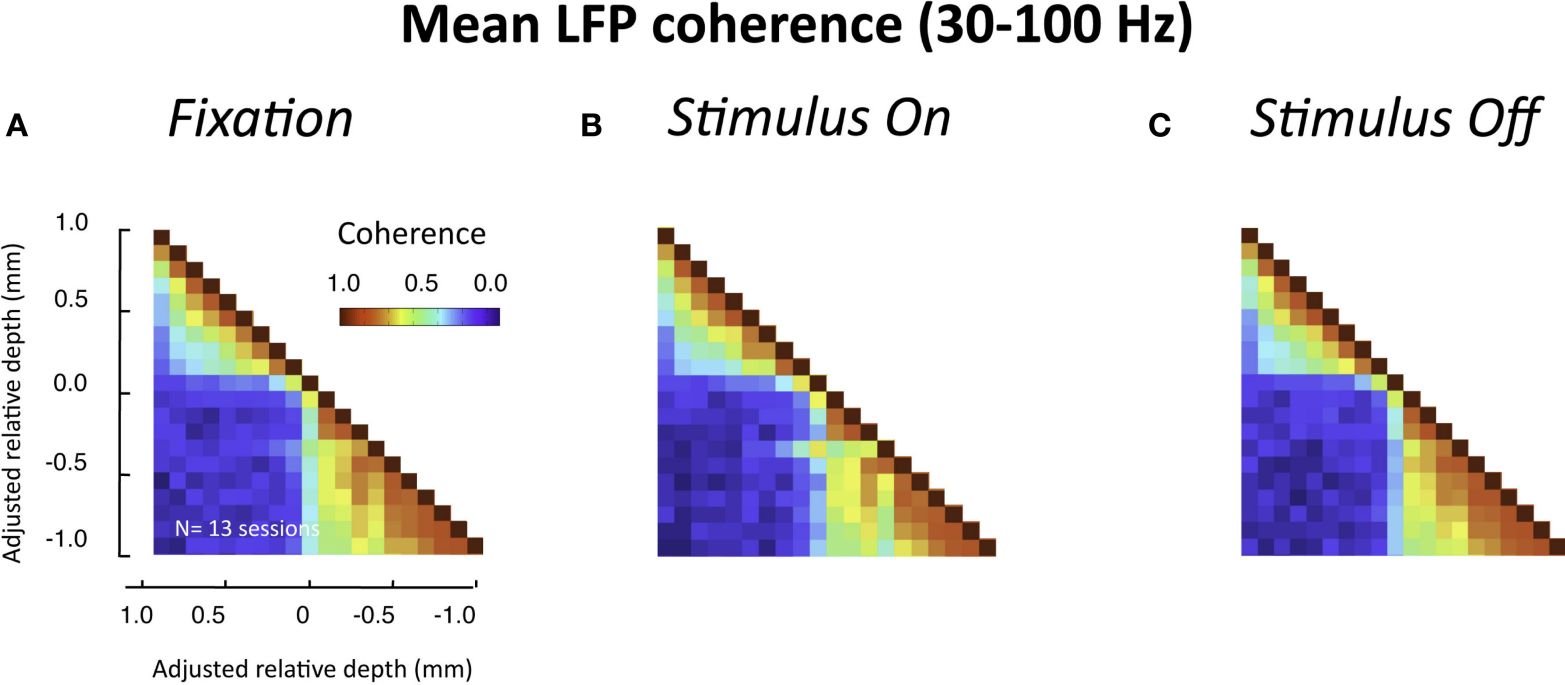
**Interlaminar coherence during visual stimulation (*n* = 13 sessions, both monkeys)**. All conventions are the same as in Figure 5B. **(A)** Coherence pattern during fixation before stimulus onset (−300 to 0 ms before stimulus onset). **(B)** Coherence pattern during sustained presentation of a luminance stimulus onto the receptive field (900–1200 ms after stimulus onset). **(C)** Coherence pattern following the removal of the stimulus (600–900 after stimulus offset). Note that despite differences in the overall coherence level compared to the resting condition (Figure 5), the spatial pattern between upper and lower layers was similar.

In the experiments described above, the monkeys were at rest, sitting drowsily in a dark room. We next asked whether a similar pattern of laminar coherence would be observed in the presence of a visual stimulus. To this end, we compared coherence before, during, and after the presentation of a luminance patch onto the receptive field of the recorded site. In contrast to the resting data, in this case the monkey was actively engaged in the task, and was required to fixate a small point in the middle of a display screen throughout each trial (see Materials and Methods and Figure S1B in Supplementary Material). The population pair-wise coherence in the gamma-range for each of these epochs is depicted in Figure 7 (all conventions are the same as in Figure 5B). We chose time windows for this analysis that minimized stimulus-related transients (i.e., >900 ms following stimulus onset and >600 ms following stimulus offset). Note that neither the task nor the stimulus significantly altered the spatial pattern of interlaminar correlation, although the overall level of LFP coherence was lower for all three conditions than during rest. The functional division into two main laminar compartments thus seems to be a fundamental principle of organization in the visual cortex, which is not disrupted by sensory activation and processing.

## Slow Power Fluctuations

**Figure 8:**
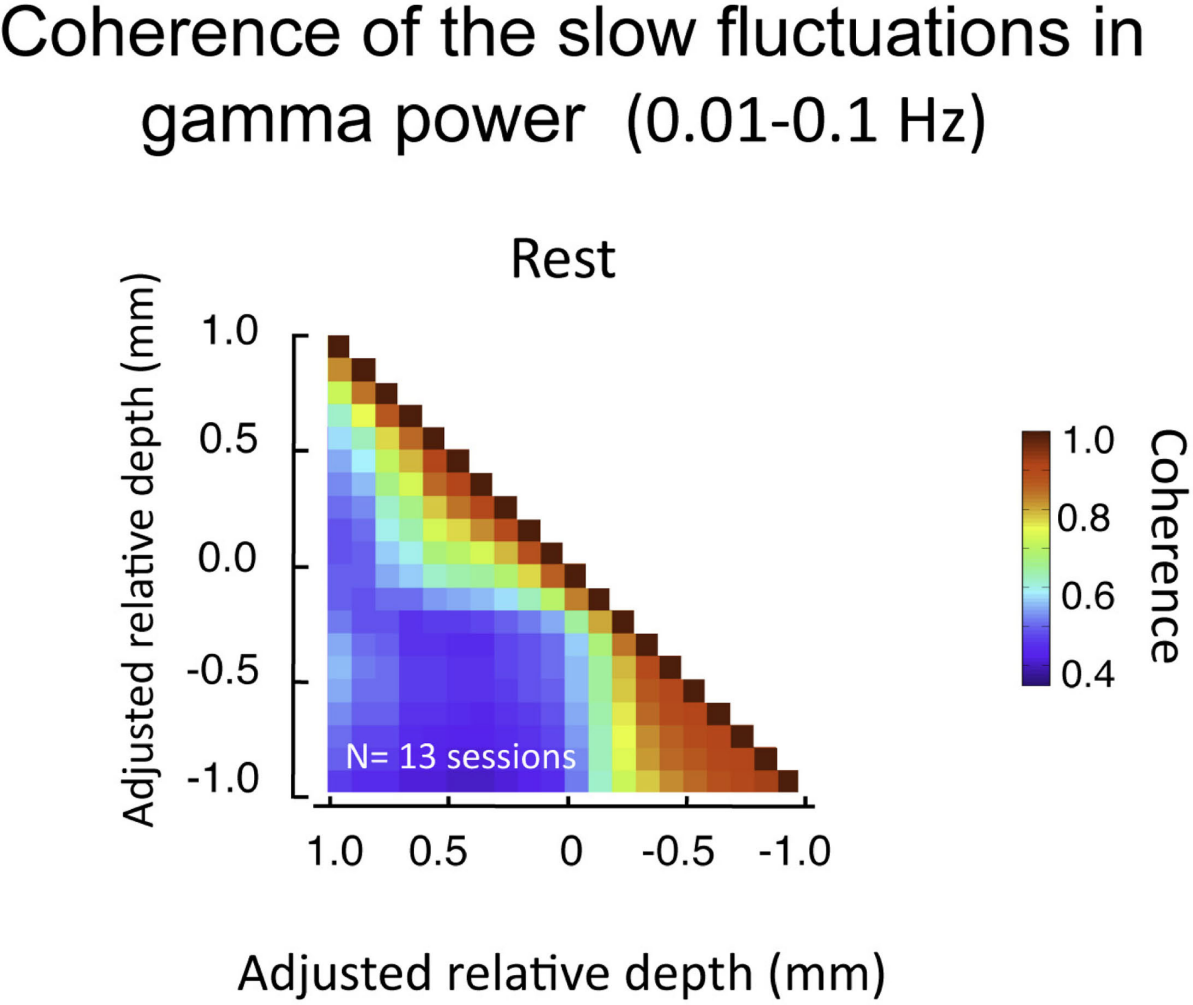
**Pair-wise coherence of the slow fluctuations in gamma power computed for all sessions (lasting 20 min each)**. Data presented in same format as Figure 7, but now pertaining to 0.01–0.1 Hz fluctuations in the magnitude of the gamma-range LFP activity. Note these fluctuations show moderate background coherence (i.e., the blue in the plot is roughly 0.5). However, as with the voltage coherence shown above, the power coherence is highest within the same laminar compartment.

The above analysis focuses on LFP fluctuations that vary on the time scale of milliseconds (in our case, filtered between 1 and 100 Hz). Another relevant signal that can be computed from the same LFP data pertains to changes that are much slower (<0.1 Hz). This signal, which we term the BLP, corresponds to magnitude of the envelope of the LFP signal filtered in a particular frequency band (Leopold et al., 2003; see Materials and Methods). The BLP signal exhibits properties that are very different from the LFP. For instance, whereas coherence in the LFP in the gamma-range falls to near zero between cortical sites separated by 2.5 mm the slow BLP shows robust coherence between recording sites separated by up to 10.6 mm(Leopold and Logothetis, 2003; Leopold et al., 2003). Based on those findings, it might be expected that the very low frequency fluctuations in the gamma BLP would be highly synchronous between all electrode contacts in the present study since they are spaced within few hundreds of microns of each other. Surprisingly, we found that, like raw LFP described above, the BLP coherence was confined to superficial and deep compartments, with much lower coherence between compartments (Figure 8). The slow BLP changes have been shown to correlate strongly with resting state fMRI fluctuations (Shmuel and Leopold, 2008; Schölvinck et al., 2010). Thus the present findings raise the question whether slow fluctuations in the upper and lower laminar zones bear a different relationship to the fMRI signal, which is a topic for future investigation.

## Discussion

Here we report a pronounced segregation in the time course of spontaneous LFP signals in the primary visual cortex of awake macaque monkeys at rest. The compartmentalization into superficial and deep layers, with the transition at or near the bottom of layer 4, may reflect known functional differences between laminae. Specifically, it is possible that the LFP activity in the superficial compartment, from which efferent projections are mainly directed to extrastriate visual areas (Felleman and Van, 1991), is primarily related to corticocortical processing. In contrast, LFP activity in the deep compartment, where efferent projections are largely directed to the lateral geniculate nucleus, pulvinar, and superior colliculus, may then be primarily related to interactions with subcortical structures. This simplified interpretation, is unlikely to be perfectly accurate, however, since the apical dendrites of infragranular neurons have abundant synapses in the supragranular layers and would therefore likely contribute to the supragranular LFP. Nonetheless, the cortical–subcortical hypothesis is a candidate that warrants further investigation.

The within- and between-compartment coherence levels differed substantially for a wide range of frequencies, from an average coherence close to 1 within compartments to close to 0 between compartments (see Figure 6). This segregation has not been previously reported, probably because the laminar distribution of SOA coherence has not been investigated in this way. A few experiments have characterized the laminar distribution of spontaneous LFP activity using other approaches. For example, a previous study focused on spontaneous alpha-rhythm oscillations in several visual areas of the monkey, and found a pronounced alpha-range peak in the coherence between the CSD and multiunit signal in the infragranular and granular, but not supragranular layers (Bollimunta et al., 2008). A different study focused on “neuronal avalanches,” which are spatiotemporal patterns of spontaneous LFP activity thought to reflect a critical state of network excitability, and found them to exist only in the superficial layers of the macaque somatosensory and motor cortex (Plenz and Thiagarajan, 2007; Petermann et al., 2009; Thiagarajan et al., 2010). Like this previous work, our findings demonstrate clear differences between LFP activity in the superficial and deep cortical layers.

## Focus on the Gamma-Range

We analyzed the gamma-range separately primarily because this range showed pronounced amplitude differences between superficial and deep layers. This frequency range is also of interest because of its relevance for cognitive function (Engel and Singer, 2001; Fries et al., 2007; Fries, 2009; Schroeder and Lakatos, 2009), and because it is thought to reflect distinct and local neural processes (Bartos et al., 2007). Note, however, that the term “gamma” denotes only a range of frequencies rather than any particular mechanism. Importantly, there was no evidence in our study that activity in the gamma-range was oscillatory or even restricted to a narrow range of frequencies.

We observed higher gamma power in the superficial layers than in the deep layers. Several anatomical correlates offer potential explanations this difference. For example, the density of synapses in macaque V1 is highest SG and G layers (O’Kusky and Colonnier, 1982). Also, the relative density of certain receptor subtypes (e.g., AMPA and GABA) in humans (Eickhoff et al., 2007) and the density of interneurons in macaques (Fitzpatrick et al., 1987) are skewed toward the SG and G layers. Since synapses, interneurons and GABA receptors are all believed to be important for the local generation of gamma (Fries et al., 2007), this anatomy may well explain the power distributions we observed. Furthermore, *in vivo* measurements have shown that the laminar density of so-called fast rhythmic bursting neurons, which have been identified as generators of persistent gamma activity *in vitro* (Cunningham et al., 2004), drop sharply in layer 5 compared to the more superficial layers (although there is also an increase in layer 6) (Cardin et al., 2005).

## Local Field Potential Coherence

The coherence measurement in the present study is sensitive to the LFP synchrony between channels. Although coherence is typically expressed as a function of frequency, it does not isolate signals that are oscillatory in nature, but is instead influenced by any type of synchrony including shared, discrete events. In fact, a wide range of neural processes could account for the distinct superficial and deep zones of coherence we measured. For one, it is interesting to speculate that neuronal avalanches mentioned above, which have been observed in the superficial, but not deep, layers of cortex (Petermann et al., 2009; Thiagarajan et al., 2010), could be a source the within-compartment coherence we measured.

It is important to note that the LFP is a differential measure, and its voltage fluctuations depend to some degree on the position of the electrical reference relative to the active electrodes. The proximity of the electrical reference affects the degree of shared temporal structure between different active electrodes, which, in turn, affects any measure of coherence. In the present study the electrode shank served as the reference, and this shank also served to electrically ground the monkey. This shank surrounded each of the active contacts and was therefore distributed throughout the cortical thickness, minimizing far-field contributions to the measured LFP, and thereby enhancing local differences. Ultimately, it would be desirable to avoid referencing issues altogether by computing either the local electric field (approximated as the first spatial derivative of the voltage along the linear array) or the CSD (approximated as the second spatial derivative of the voltage multiplied by the tissue conductivity). The CSD is thought to reflect synchronous synaptic currents transferred between extracellular and intracellular compartments, and is thereby a step closer than the LFP to the generative neural processes. However, the low SNR of the CSD signal poses a challenge for the type of analysis used in this study, in which signals cannot averaged over many trials.

## Relationship to Anatomical Architecture

The results described in this study may ultimately shed light on structure-function relationships in the visual cortex. The primary visual cortex differs from other visual and non-visual areas in several key aspects of its cytoarchitecture, including its laminar makeup, including prominent extent of layer 4 compared to other visual areas (Lund, 1988), along with its idiosyncratic microvasculature (Weber et al., 2008). Recent reports find LFP differences between V1 and higher visual areas including laminar differences in the gamma frequency range during cognitive tasks (Buffalo et al., 2004; Chalk et al., 2010). V1 has a laminar distribution of neurotransmitter receptors that distinguish it from other areas (Eickhoff et al., 2007), including cholinergic receptors (Disney and Aoki, 2008), which are thought to play a role in shaping activity in the gamma frequency band of the LFP (Munk et al., 1996; Fisahn et al., 1998). In the future, a wider sampling of cortical areas using the techniques described here may be useful to gain a deeper understanding of the link between cortical laminar structure and neurophysiological function.

## Conflict of Interest Statement

The authors declare that the research was conducted in the absence of any commercial or financial relationships that could be construed as a potential conflict of interest.

## Supplementary Material

The Supplementary Material for this article can be found online at http://www.frontiersin.org/neuroscience/systemsneuroscience/paper/10.3389/fnsys.2010.00031/
